# The Emerging Role of Exosomes in the Treatment of Human Disorders With a Special Focus on Mesenchymal Stem Cells-Derived Exosomes

**DOI:** 10.3389/fcell.2021.653296

**Published:** 2021-07-07

**Authors:** Soudeh Ghafouri-Fard, Vahid Niazi, Bashdar Mahmud Hussen, Mir Davood Omrani, Mohammad Taheri, Abbas Basiri

**Affiliations:** ^1^Department of Medical Genetics, School of Medicine, Shahid Beheshti University of Medical Sciences, Tehran, Iran; ^2^Department of Tissue Engineering and Applied Cell Sciences, School of Advanced Technologies in Medicine, Shahid Beheshti University of Medical Sciences, Tehran, Iran; ^3^Department of Pharmacognosy, College of Pharmacy, Hawler Medical University, Erbil, Iraq; ^4^Skull Base Research Center, Loghman Hakim Hospital, Shahid Beheshti University of Medical Sciences, Tehran, Iran; ^5^Urology and Nephrology Research Center, Shahid Beheshti University of Medical Sciences, Tehran, Iran

**Keywords:** extracellular medicine, regenerative medicine, mesenchymal stem cells, biomarkers, expression

## Abstract

Extracellular vesicles (EVs) are produced by diverse eukaryotic and prokaryotic cells. They have prominent roles in the modulation of cell-cell communication, inflammation versus immunomodulation, carcinogenic processes, cell proliferation and differentiation, and tissue regeneration. These acellular vesicles are more promising than cellular methods because of the lower risk of tumor formation, autoimmune responses and toxic effects compared with cell therapy. Moreover, the small size and lower complexity of these vesicles compared with cells have made their production and storage easier than cellular methods. Exosomes originated from mesenchymal stem cells has also been introduced as therapeutic option for a number of human diseases. The current review aims at summarization of the role of EVs in the regenerative medicine with a focus on their therapeutic impacts in liver fibrosis, lung disorders, osteoarthritis, colitis, myocardial injury, spinal cord injury and retinal injury.

## Introduction

Being released from diverse eukaryotic and prokaryotic cells, extracellular vesicles (EVs) have prominent roles in the modulation of cell-cell communication, inflammation versus immunomodulation, carcinogenic processes, cell proliferation and differentiation, and tissue regeneration (Soleymani-Goloujeh et al., 2018). Collectively, EV include an assorted cell-secreted assemblies enclosed by a bilayer phospholipid membrane containing various macromolecules such as proteins, lipids, and nucleic acids (Robbins and Morelli, 2014; Fuster-Matanzo et al., 2015). The interaction between EVs and target cells is accomplished via various routes including the interplay between transmembrane proteins on EVs and cellular surface receptors and induction of certain signaling pathways (Raposo and Stoorvogel, 2013). Alternatively, EVs can directly fuse with their target cells and release their constituents into the cytosol after endocytosis (Raposo and Stoorvogel, 2013). Being implicated in a wide range of pathophysiological processes, EVs can be used as biomarkers of diverse disorders and targets for the design of new cell-free therapeutic options (Fuster-Matanzo et al., 2015). Microvesicles and exosomes comprise two main categories of EVs with sizes about 100 nm^–1^ μm and 30–150 nm, respectively (Doyle and Wang, 2019).

Due to the heterogeneity of EVs and their sizes, isolation, identification and classification of EVs are challenging issues (Ramis, 2020). Yet, ample works are being conducted to enhance procedures for investigation of EVs. A new aqueous two-phase system-based method has been established for highly efficient isolation of EVs with high level of purity (Kırbas̨ O. K. et al., 2019). Another EV immunolabeling method has been introduced that can be incorporated into the currently used nanoparticle tracking analysis protocols to provide particle concentration, size scattering, and surface characteristics of EVs (Thane et al., 2019). Moreover, a luminescence-based assay has been developed that can obviously discriminate between EV uptake and EV binding to the surface of target cells (Toribio et al., 2019). Lastly, generation of an inducible CD9-GFP mouse has provided a method for EV labeling in a cell-type specific way and simultaneous analysis of EVs *in vivo* (Neckles et al., 2019). It is worth mentioning that the method used for isolation of EVs has clear impact on the integrity and purity of EVs.

Several studies have emphasized on the role of EVs in tissue engineering and regenerative medicine with the aim of reestablishment of an injured or abnormal-working tissue (De Jong et al., 2014). The current review aims at summarization of the role of EVs in the regenerative medicine with a focus on their therapeutic impacts in liver fibrosis, lung disorders, osteoarthritis, colitis, myocardial injury, spinal cord injury and retinal injury.

## Liver Fibrosis

Mesenchymal stem cells (MSCs) has been introduced as therapeutic option for liver disease based on their ability to differentiate into hepatic cells and their aptitude in the reduction of inflammatory responses through secretion of certain anti-inflammatory cytokines (Lou et al., 2017a). MSC-derived exosomes are superior to MSCs regarding lower probability induction of tumors, rejection and toxicity (Lou et al., 2017a). Expression of miR-122 has been decreased in transactivated hepatic stellate cells (HSCs). Exosomes originated from adipose tissue-derived MSCs have been displayed to up-regulate miR-122. Up-regulation of miR-122 has suppressed the proliferation of LX2 cells through targeting P4HA1 gene. This miRNA has been shown to reduce collagen maturation and extracellular matrix synthesis (Li J. et al., 2013). MSC-derived substances might also be used in the treatment of fulminant hepatic failure (FHF). In a rat model of acute hepatic injury, systemic administration of MSC-conditioned medium has enhanced survival of animals, prevented the production of hepatic damage markers, decreased apoptosis rate of hepatic cells and increased the quantities of proliferating hepatic cells. Taken together, MSC-conditioned medium has direct anti-apoptotic and pro-mitotic impacts on hepatic cells and is a possible method for the management of FHF (Van Poll et al., 2008). Besides, MSC-conditioned medium (CM) has been revealed to influence apoptotic processes in cultured mouse primary hepatic cells following induction of hepatic injury using carbon tetrachloride (CCl4). In this study, bone marrow MSCs (BM-MSCs) have been used for generation of CM. Authors have demonstrated up-regulation of IL-6 in the CCl4-CM treated hepatocytes on the first day of culture. Moreover, levels of fibroblast-like-protein (FGL1) have been increased after 48 h, while annexin V positive hepatocytes have been decreased at day 3 post plating. These results have indicated the impact of this CM in attenuation of CCl4-induced apoptosis in liver cells via induction of FGL1 (Xagorari et al., 2013). Another study has assessed the impact of MSCs on the phenotype and activity of natural killer T (NKT) cells in a mouse model of hepatic injury induced by concanavalin A and α-galactosylceramide. *In vitro* culture of liver NKT cells with MSCs has resulted in production of lower quantities of TNF-α, IFN-γ and IL-4 proinflammatory cytokines while over-production of the anti-inflammatory cytokine IL-10 upon stimulation with α-galactosylceramide. MSCs have also deceased levels of apoptosis-inducing ligands on hepatic NKT cells and diminished levels of pro-apoptotic genes in the hepatic tissue. Notably, MSCs have decreased the cytotoxic effects of hepatic NKT cells against hepatocytes. These effects have been shown to be mediated by indoleamine 2,3-dioxygenase (IDO) and inducible nitric oxide synthase (iNOS). Moreover, human MSCs have also been shown to reduce release of proinflammatory cytokines in α-galactosylceramide-stimulated human peripheral blood mononuclear cells via a similar route and decrease their cytotoxic effects against hepatic cells (Gazdic et al., 2018). In addition, transplantation of human umbilical cord-MSCs (UC-MSCs) into acutely damaged and fibrotic liver have restored hepatic function and ameliorated liver fibrosis. Exosomes originated from these cells have decreased the surface fibrous capsules, lessened inflammatory responses in the hepatic tissue and collagen deposition in CCl4-associated fibrotic liver. Levels of collagen type I and III, TGF-β1 and phosphorylated Smad2 have also been decreased (Li T. et al., 2013). Table 1 reviews the results studies which reported the role of extracellular vesicles in the treatment of liver disorders.

**TABLE 1:** Summary of studies which reported the role of extracellular vesicles in the treatment of liver disorders.

**Cell origin**	**Type of secreted vesicle**	**Disease**	**Target cells or tissues**	**Molecular mechanism**	**Biological effect and therapeutic applications**	**References**
CP-MSCs	Exosome	liver fibrosis	Hepatocytes	microRNA-125b	Increase liver Regeneration by inhibition of hedgehog (Hh) signal	Hyun et al., 2015
βMSCs	Exosome	CLP	Hepatocytes	miR-146a	Diminish liver damage and decrease mortality	Song et al., 2017
BM-MSCs	Conditioned medium	Acute liver failure	Th1 and Th17 cells	IL-10; CXCR3 and CCR5	Decrease invasion in the injured liver	Van Poll et al., 2008
HA-MSCs	EVs	Acute liver failure	Hepatocytes	lncRNA H19	Increase hepatocytes proliferation and decrease mortality	Jin et al., 2018
HA-MSCs	Exosome	Acute liver failure	Macrophages	miR-17	Suppress the activation of NLRP3 inflammatory bodies	Liu et al., 2018
UC-MSCs	Exosome	Liver fibrosis	Hepatic cells	TGF-β/Smad2	Decrease collagen production	Li T. et al., 2013
hUCMSCs	Exosome	Acute liver failure	Hepatocytes	miR-299-3p	Decrease inflammation through suppression of NLRP3-related pathways	Zhang et al., 2020
MSC	Exosome	HBV	Macrophage	HBV-miR-3/SOCS5/STAT1	Macrophage M1 polarization and IL-6 secretion	Zhao X. et al., 2020
MSC	Exosome	HBV	Macrophage	HBV-infected hepatocyte exosomes/MyD88, TICAM-1, and MAVS	Enhance immune response in the host	Kouwaki et al., 2016
BM-MSCs	Conditioned medium/Exosome	Acute liver failure	Hepatocytes	IDO-1/KYN; HGF; FLP1; IL-6/gp130; Bcl-xL; Cyclin D1	Increase proliferation and suppress apoptosis	Xagorari et al., 2013; Gazdic et al., 2018; Milosavljevic et al., 2018
hUCMSCs	Exosome	Liver failure	Hepatocytes	GPX1	Decrease oxidative stress and apoptosis	Yan et al., 2017
BM- MSCs	Exosome	Autoimmune hepatitis	Hepatocytes	miR-223	ALT and AST levels were diminished and apoptosis was inhibited.	Chen et al., 2018
BM-MSCs	Conditioned medium	Acute liver failure	Natural killer T cells	IDO-1/KYN	Decrease inflammatory Cytokines secretion and decrease cytotoxicity	Xagorari et al., 2013; Gazdic et al., 2018; Milosavljevic et al., 2018
MSC	Exosome	NAFLD	Macrophage	miR122-5p/lysosome	M1 polarization	Zhao Z. et al., 2020
MSC	Exosome	Hepatocellular carcinoma	Macrophage	lncRNA TUC339/Toll-like receptor signaling and FcgR-mediated phagocytosis	Decrease in pro-inflammatory cytokine secretion and enhance the phagocytosis	Li X. et al., 2018
MSC	Exosome	Hepatocellular carcinoma	Macrophage	Exo-con/STAT3	Enhance cytokine secretion in macrophages	Cheng et al., 2017
BM-MSCs	Exosome	Acute liver failure/liver fibrosis	Leukocyte	IDO-1/KYN; TGF-; IL-10	Suppressed activation of the inflammasome	Lou et al., 2017a; Milosavljevic et al., 2017
MSC	Exosome	Alcoholic liver disease	Macrophage	miR-27A/CD206 on monocytes	M2 polarization	Saha et al., 2016
MSC	Exosome	Alcoholic liver disease	Macrophage	CD40L/Caspase-3	M1 polarization	Eguchi et al., 2016
MSC	Exosome	Alcoholic liver disease	Monocytes	miR-122/HO-1	Increase sensitivity of monocytes to LPS	Fairclough et al., 2014
MSC	Exosome	Alcoholic liver disease	Kupffer cells	Mitochondrial double-stranded RNA/TLR3 in Kupffer cells	Increase in IL-1b and IL–17A levels	Lee et al., 2020
MSC	Exosome	NAFLD	Macrophage	Hepatocyte-derived EV/DR5/Caspase/ROCK1	Enhance macrophage pro-inflammatory	Hirsova et al., 2016
MSC	Exosome	NAFLD	Monocytes	Lipotoxic EVs/ITGb1	Increase monocyte adhesion and inflammatory response	Gallina et al., 2019
MSC	Exosome	Hepatocellular carcinoma	Macrophage	miR-23a-3p/PTEN/AKT	Inhibition of T-cell function	Li T. et al., 2013
MSC	Exosome	Hepatocellular carcinoma	Hepatocytes	miR-142-3p/RAC1	supress hepatocellular carcinoma cell migration and invasion	Zhang et al., 2014
UC- MSCs	EVs	Hepatitis	Liver cells	miR-let-7f, miR-145, miR-199a, miR-221	Protect liver cells against HCV	Qian et al., 2016
BM-MSCs	Exosome	Liver injury	–	Cationized pullulan	Anti-inflammatory effect	Tamura et al., 2017
MenSCs	Exosome	Fulminant liver failure	Hepatocytes	ICAM-1, osteoprotegerin, angiogenin-2,	Decrease mortality and inhibits apoptosis	Yang et al., 2017a
HA-MSCs	Exosome	liver fibrosis	Hepatocytes	miR-122	Decrease collagen deposition	Lou et al., 2017b
MSC	Exosome	HCV	Macrophage	Anti-HCV miRNA-29/TLR3-activated macrophages	Decrease HCV replication	Zhou et al., 2016
MSC	Exosome	HCV	Monocytes	Exosome-packaged HCV/TLR7/8	Differentiation of monocytes into macrophages	Saha et al., 2017
MSC	Exosome	Alcoholic liver disease	Macrophage	miR-155/Hsp90	Enhance in inflammatory macrophages	Babuta et al., 2019
MSC	Exosome	NAFLD	Macrophage	mi R-192-5p/Rictor/Akt/FoxO1	M1 polarization	Liu et al., 2020

*BM-MSCs, bone marrow mesenchymal stem cells; UC-MSCs, umbilical cord mesenchymal stem cells; KYN, Kynurenine; CCR5, C-C chemokine receptor type 5; TGF-β, transforming growth factor beta; IDO-1, indoleamine 2,3 dioxygenase-1; HA-MSC, human adipose-derived mesenchymal stem cells; MenSC-Exos, human menstrual blood stem cell-derived exosomes; hUCMSC-Exos, Human umbilical cord mesenchymal stem cell-derived exosomes; CP-MSC, chorionic plate-derived mesenchymal stem cells; βMSC, MSC pre-treated with IL-1β; CLP, Puncture induced sepsis; NAFLD, Nonalcoholic fatty liver disease.*

## Lung Disorders

Exosomes originated from endothelial progenitor cells (EPCs) have been shown to preclude sepsis-associated endothelial dysfunction and lung damage partly because of the presence of miR-126 in these vesicles (Zhou et al., 2018). Moreover, intratracheal injection of EPC-derived exosomes has been shown to ameliorate lung damage following lipopolysaccharide-induced acute lung injury. This type of treatment has also decreased cell quantities, protein amounts, and cytokine levels in the bronchoalveolar lavage fluid, representing a decrease in permeability and inflammatory responses possibly through a miR-126-dependent mechanism. Similarly, up-regulation of miR-126-3p in human small airway epithelial cells has been shown to affect expression of PIK3R2, miRNA-126-5p has been shown to suppress expression of HMGB1 and VEGFα which are involved in the regulation of inflammatory responses and permeability, respectively. Notably, both miRNAs enhance the levels of tight junction proteins proposing a possible mechanism through which miR-126 alleviates LPS-induced lung damage (Zhou et al., 2019). Another study has demonstrated the efficacy of CM or EVs originated from BM-MSCs in amelioration of inflammation in an animal model of mixed Th2/Th17, neutrophil-associated allergic airway inflammation. Systemic injection of both CM and EVs isolated from human and murine MSCs, at the commencement of antigen challenge in formerly sensitized animal models has considerably amended the airway hyperreactivity, inflammatory reactions in lung, and the antigen-specific CD4 T-cell Th2 and Th17 phenotype (Cruz et al., 2015). Adipose tissue-derived MSCs and EVs have been shown to act in a different way on static lung elastance, regulatory T cells and CD3+CD4+ T cells of bronchoalveolar lavage fluid, and production of proinflammatory cytokines. Yet, their effects on reduction of eosinophils in lung tissue, content of collagen fibers in airways and lung parenchyma, production of TGF-β in lung tissue, and thymic CD3+CD4+ T cells have been similar (de Castro et al., 2017). Supplementary Table 1 gives a summary of studies which reported the role of EVs in the treatment of lung disorders.

## Osteoarthritis

Mao et al. have demonstrated elevated exosomal levels of miR-92a-3p in the chondrogenic exosomes of MSCs despite its low levels in the osteoarthritis chondrocyte-originated exosomes. Notably, MSC-miR-92a-3p exosomes have stimulated cartilage proliferation and increased expressions of matrix genes in an MSC model of chondrogenesis and in primary human chondrocytes, respectively. miR-92a-3p has been shown to suppress expression of WNT5A in both models. Moreover, MSC-miR-92a-3p exosomes have inhibited cartilage destruction in the mouse model of osteoarthritis (Mao et al., 2018). Zhu et al. have shown the effects of exosomes originated from synovial membrane MSCs as well as exosomes of MSCs derived from iPSCs in the attenuation of osteoarthritis in an animal model of this disorder. Yet, the latter exosomes have had a greater therapeutic impact. Both types of exosomes have also enhanced chondrocyte migration and proliferation with those secreted by MSCs derived from iPSCs being superior to the other (Zhu et al., 2017). Supplementary Table 2 gives a summary of studies which reported the role of EVs in the treatment of osteoarthritis.

## Colitis

Microvesicles containing miR-200b have been shown to amend the abnormal morphology of TGF-β1-treated intestinal epithelial cells and recover the 2,4,6-trinitrobenzene sulfonic acid-induced fibrosis in the colon possibly through inhibition of epithelial-mesenchymal transition (EMT) and mitigation of fibrosis. These effects have been accompanied by over-expression of E-Cad, and down-regulation of vimentin, α-SMA, ZBE1, and ZEB2 (Yang et al., 2017b). Mao et al. have appraised the impact of human UC-MSCs-derived exosomes in an animal model of dextran sulfate sodium- induced inflammatory bowel disease (IBD). These exosomes have been shown to relieve IBD course through enhancing IL-10 level while decreasing TNF-α, IL-1β, IL-6, iNOS, and IL-7 levels. Besides, treatment with these exosomes has led to reduction of macrophage infiltration into the colon (Mao et al., 2017). BM-MSC-derived EVs have also had beneficial effects in an animal model of 2,4,6-trinitrobenzene sulfonic acid-induced colitis when injected intravenously. These effects are possibly mediated through down-regulation of NF-κBp65, TNF-α, iNOS, and COX-2 in damaged colon. Moreover, these vesicles have remarkably decreased IL-1β and increased IL-10 levels. In addition, BM-MSC-derived EVs have been shown to modulate the anti-oxidant/oxidant equilibrium, and moderate apoptotic pathways (Yang et al., 2015). Supplementary Table 3 gives a summary of studies which reported the role of EVs in the treatment of colitis.

## Myocardial Injury

Mesenchymal stem cell-derived exosomes have been shown reduce the size of infarct in a mice model of myocardial ischemia/reperfusion injury thus being implicated in the tissue repair (Lai et al., 2010). MSCs have also been demonstrated to suppress myocardial cell apoptosis and enhance regenerative process in the endothelial cell microvasculature through production of exosomes. SDF1 has been identified as the effective exosome ingredient which has protective effects on cardiac function and suppresses myocardial injury (Gong et al., 2019). Akt-containing exosomes have improved cardiac function in an animal model of acute myocardial infarction. These vesicles could accelerate proliferation and migration of endothelial cells and construction of tube-like configurations and blood vessels *in vitro* and *in vivo*, respectively. These effects have been mediated through up-regulation of PDGF-D (Ma et al., 2017). Supplementary Table 4 provides a summary of studies which reported the role of EVs in the treatment of cardiac disorders.

## Spinal Cord Injury

Bone marrow-MSC-derived EVs have been reported to decrease brain cell death, increased survival of neurons and improved regenerative processes and motor function. In addition, these vesicles has attenuated blood-spinal cord barrier and reduced pericyte coverage in the animal models of spinal cord injury. Exosomes have been shown to decrease pericyte migration through inhibition of NF-κB p65 signaling and reduction of the permeability of the blood-spinal cord barrier (Lu et al., 2019). miR-133b has been identified as an important ingredient of MSC-derived exosomes. Administration of miR-133b-containing exosomes has enhanced the recovery of hindlimb function in an animal model of spinal cord injury. Moreover, these exosomes have decreased lesion size, protected neurons, and stimulated regenerative processes of axons RhoA has been identified as a direct target of miR-133b. miR-133b-containing exosomes could activate neuron survival pathways such as ERK1/2, STAT3, and CREB (Li D. et al., 2018). Systemic injection of MSCs-derived exosomes has also been shown to reduce lesion dimension and enhance functional recovery after induction of spinal cord injury in animal models. These exosomes have also decreased cell apoptosis and inflammatory responses in the damaged spinal cord as evidenced by reduction of expressions of pro-apoptotic protein and TNF-α and IL-1β proinflammatory cytokines while increased levels of BCL2 and IL-10. MSCs-derived exosomes have also increased angiogenic processes (Huang et al., 2017). Supplementary Table 5 has shown summary of studies which reported the role of extracellular vesicles in the treatment of spinal cord injury.

## Other Disorders

The beneficial effects of EVs in the treatment of several other disorders such as renal fibrosis, stroke, neurodegenerative disorders and retinal injury have been assessed in independent studies. For instance, experiments in an animal model of middle cerebral artery occlusion have shown the impact of MSCs in enhancement of miR-133b expression in the ipsilateral hemisphere. *In vitro*, expression of this miRNA has been increased in MSCs and in MSC-derived exosomes after exposure to ipsilateral ischemic tissue extracts. Expression of miR-133b has also been augmented in primary cultured neurons and astrocytes exposed with the exosome-enriched materials produced by these MSCs. The results of this study indicates communication between MSCs and brain parenchymal cells and the impact of such interplay on regulation of neurite outgrowth through exosome-mediated transfer of miR-133b to neural cells (Xin et al., 2012). The beneficial effects of BM-MSC-derived EVs have also been assessed in Alzheimer’s disease. Cui et al. have shown improvement of some neurological abnormalities in an animal model of this disorder following administration of MSC-derived exosomes. Administration of normoxic MSCs-derived exosomes has amended cognition and memory deficits, decreased plaque deposition and brain Aβ amounts. These effects are associated with down-regulation of TNF-α and IL-1β and up-regulation of IL-4 and IL-10. Exosomes from hypoxia-preconditioned MSCs have exerted superior effects in learning and memory abilities and plaque deposition and Aβ amounts (Cui et al., 2018). Another experiment in an animal model of glaucoma induced by chronic ocular hypertension has shown neuroprotective effect of BM-MSC-derived EVs and reduction of the quantity of degenerating axons in the optic nerve (Mead et al., 2018). In addition, BM-MSCs have been shown to extend the survival of allogenic renal transplant in animal models. Mechanistically, these cells increase miR-146a levels in dendritic cells of the treated animals. Similarly, BM-MSC-derived microvesicles enhance miR-146a levels in both immature and mature dendritic cells *in vitro*, while decreasing IL-12 levels in mature dendritic cells. Therefore, BM-MSCs-originated microvesicles enhance outcome of allogenic renal transplantation via suppression of dendritic cell maturity by miR-146a (Wu et al., 2017). Supplementary Table 6 summarizes the results of studies which reported the role of EVs in the treatment of various disorders.

## Discussion

Extracellular vesicles are beneficial tools of delivery of biomolecules in the field of regenerative medicine. These acellular vesicles are more promising than cellular methods because of the lower risk of tumor formation, autoimmune responses and toxic effects compared with cell therapy (Katsuda et al., 2013a). Moreover, the small size and lower complexity of these vesicles compared with cells have make their production and storage easier than cellular methods (Katsuda et al., 2013a).

**FIGURE 1:**
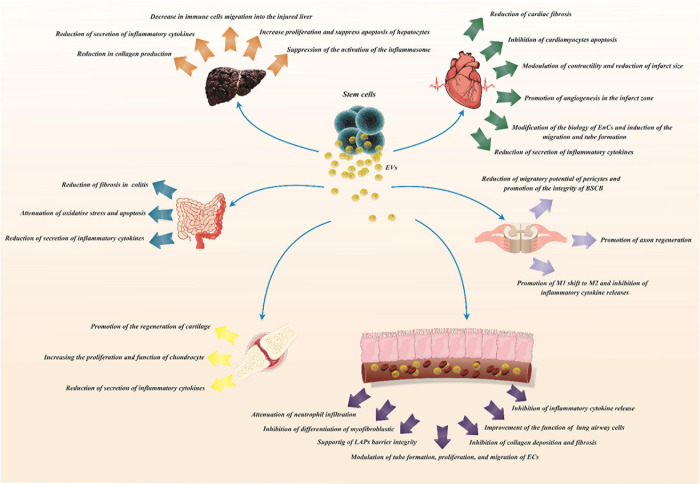
Role of MSC-derived extracellular vesicles in the regeneration of different tissues.

Mesenchymal stem cells have been suggested as the most favorable source for cell-based therapy due to their multi-lineage differentiation capacity and immuno-modulatory features (Harrell et al., 2019). As MSCs have therapeutic application in the prevention of parenchymal cell defects and enhancement of tissue regeneration in animal models of myocardial injury, renal failure, stroke and other disorders, the effects of MSC-derived EVs in the treatment of these disorders have been assessed reporting promising results. Figure 1 illustrates role of these particles in regeneration of different tissues.

Proliferation, survival, apoptosis and senescence of MSCs might be affected by EVs. Endothelial cell-derived exosomes have been found to induce angiogenesis through suppression of cell senescence. Moreover, transfer of miR-214 by these vesicles has decreased expression of ATM gene in recipient cells, reducing their senescence (van Balkom et al., 2013). Further evidence for modulation of apoptosis by EVs has come from studies that revealed the presence of anti-apoptotic miRNAs in exosomes originated from human cardiac progenitor cells as well as bone marrow MSCs (Reis et al., 2012; Barile et al., 2014).

Conditioned medium or exosomes originated from MSCS can prevent liver injury through different mechanisms such as modulation of immune responses, induction of immune tolerance via affecting IDO and iNOS levels and changing expression of a number of miRNAs. In animal models of acute lung injury, administration of EPC-derived EVs has ameliorated tissue damage particularly through their cargo miR-126. Besides, a growing experience demonstrates beneficial effects MSC-based cell therapies in animal models of asthma suggesting a novel strategy for treatment of severe refractory asthma (Cruz et al., 2015).

The underlying mechanism of beneficial effects of MSC-derived EVs in the regeneration of tissues and inhibition of tissue damage has been verified in a number of studies through assessment of the cargo of EVs. However, the synergic effects of EV ingredients should not be ignored as these acellular particles contain several agents which might affect cellular processes via different routes. Moreover, EVs have several target cells in the microenvironment; therefore can affect the function of various cells such as endothelial cells, epithelial cells and different immune cells. The cell-specific functions of EVs should be also assessed in order to design the most appropriate therapeutic modalities.

The long half-life of exosomes and their ability in penetrating cell membranes and targeting specific kinds of cells have potentiated these vesicles as candidates for therapeutic applications. Moreover, the fact that exosomes are not perceived by immune system as foreign bodies makes them more appropriate for the these applications (Lai et al., 2013).

The efficacy of EVs originated from adipose tissue-MSCs in the amelioration of clinical and pathological features in animal models of disorders has indicated the vast source of finding MSCs and their related biomaterials, thus improving the applicability of these modalities in several settings. Exosomes secreted by iMSC might also have appropriate therapeutic impact in certain conditions due to their inexhaustible potential. Besides, microvesicles can be used for transferring certain cargo from genetically modified stem cells to target cells. Due to stability of exosomes in the circulation, systemic administration of these vesicles is an efficient method for transferring their cargo to target cells.

A challenge in the field of application of MSCs in the regenerative medicine has arisen from the observed different effects of some MSC-derived EVs and MSCs on molecular targets, biomolecules and tissue construction which necessitate precise assessment of the pathways targeted by each modality.

Taken together, EVs have emerged as potential vehicles for amelioration of damaged tissues and improvement of tissue organization. However, the molecular mechanisms of EVs-induced changes in tissues should be appraised further. Moreover, the majority of studies have been conducted in experimental models. Therefore, applicability of these techniques in medical practice must be more comprehensively assessed. Besides, understanding the cargo trafficking pathways of EVs is necessary to control the cargo of EVs and avoid unspecific effects. Lack of knowledge in these fields has limited application of EVs in treatment of human disorders. Finally, lack of segregation of the therapeutic effects of “cells” versus “cell-derived EVs” is a limitation of a number of studies in this field.

## Author Contributions

MT and SG-F wrote the draft and revised it. AB, MO, and VN designed the tables, figures, and collected the data. All authors approved submitted version.

## Conflict of Interest

The authors declare that the research was conducted in the absence of any commercial or financial relationships that could be construed as a potential conflict of interest.
