# Supplementary material for: The Emerging Role of Exosomes in the Treatment of Human Disorders With a Special Focus on Mesenchymal Stem Cells-Derived Exosomes
Source: Front Cell Dev Biol. 2021 Jul 7;9:653296. doi: 10.3389/fcell.2021.653296 (PMC8293617; doi:10.3389/fcell.2021.653296)
Supplement: Supplementary file 1 [file Data_Sheet_1.docx]

Supplementary Table 1. Summary of studies which reported the role of extracellular vesicles in the treatment of lung disorders.

| Cell origin | Type of secreted vesicle | Disease | Target cells or tissues | Molecular mechanism | Biological effect & therapeutic applications | Reference |
| --- | --- | --- | --- | --- | --- | --- |
| EPC | Exosome | ALI | HSAEpC | miR-126-3p and miR-126-5p | Maintained LAPs barrier integrity and decreased infiltration of neutrophil | (49) |
| EPC | Exosome | ALI | ECs | miR-126 | Modulated tube Formation, proliferation, and migration ECs. | (52) |
| HA-MSC | EVs | ALI | Lung tissue | ↓TNF-α, ↓ROS | Decrease pro-inflammatory  cytokines and enhance anti-inflammatory cytokines | (53) |
| HA-MSC | EVs | ALI | Lung tissue | ↓IL-6, ↓TRPV4, ↑β-Catenin,  ↑VE-Cadherin ↓MPO | Diminishing of pulmonary endothelial barrier permeability and decrease pro-inflammatory  cytokines | (54) |
| HA-MSC | EVs | ALI | Lung tissue | ↓IL-1β and ↑IL-10 | Decrease neutrophil and  macrophage recruitment in alveolar fluid | (55) |
| EPC | Exosome | CLP-induced  sepsis | ECs | miR-126-3p  and miR-126-5p | Improved survival of mice suffering from lung injury | (48) |
| AT-MSCs/BM-MSCs | Exosome / conditioned medium | Asthma | Neutrophils/ eosinophils/ Th2 and  Th17 cells/ | IL-10 | Inhibited secretion of cytokine | (50, 51) |
| BM-MSCs | Exosome | Asthma | DCs | IL-10; TGF- | Decreased antigen  presenting function | (56) |
| BM-MSC | EVs | ALI | BAL | ↓MIP-2, ↓TNF-α, ↑LTB4 | Increase monocyte phagocytosis and  decreased leukocytes and neutrophils levels | (57) |
| Placental chorionic and  decidual MSCs | EVs | ALI | Lung tissue | ↓TNF-α | Decrease in pro-inflammatory  cytokines; enhance anti-inflammatory cytokines;  enhancement of  epithelial cell migration | (58) |
| BM-MSC | EVs | ALI | Lung tissue | ↓MPO, ↓IL-1β, ↓TNF-α, ↓IL-6 | Decrease in pro-inflammatory  cytokines; enhance anti-inflammatory cytokines | (59) |
| iPSC | iPSC CM | ALI | - | Decreased the activity and levels of NFκβ and TNF-α and IL-6 | Attenuated the course of endotoxin-associated ALI | (60) |
| ESC-M  SC | ESC  Derived  MSC | ALI | - | Reduced the secretion of Macrophage-inflammatory protein 2 and TNF-α | Attenuated lung injury and inflammation | (61) |
| BM-MSC | EVs | Fibrosis | Lung tissue | ↓Arg-1, ↓CCL2 | Decrease in pro-inflammatory  cytokines; enhance anti-inflammatory cytokines; diminishing of alveolar epithelial apoptosis | (62) |
| UC-MSC | EVs | ALI | Lung tissue | Unknown mechanism | Decrease alveolar protein permeability | (63) |
| BM-MSC | EVs | ALI | Lung tissue | ↓P2X7, ↓MDA, ↓H2O2, ↑GSH | Decrease alveolar wall thickness and pro-inflammatory cytokines | (64) |
| ESC | ESC | ALI | Lung  epithelial  cells | Secretion of TGF-β, FGF, and VEGF | Attenuated lung injury and inflammation | (65) |
| BM-MSC | Exosome | Covid-19 | Lung tissue | Exosome secretom | Improve lung oxygenation | (66) |
| BM-MSCs | EVs | Idiopathic pulmonary fibrosis | Fibroblasts | miR-630 | Inhibited differentiation of myofibroblastic | (67, 68) |
| BMDMC | BMDMC | ALI | - | Suppression of  glycogen synthase kinase-3β | Attenuated lung injury, protected against early organ damage resulted from severe hemorrhagic chock | (69) |
| BM-MSC | EVs | ALI | Lung tissue | TNF-α | Antimicrobial effects;  Diminishing of pulmonary endothelial permeability | (70) |
| Amnion epithelial cells | EVs | Fibrosis | Lung tissue | ↓TGF- β | Decrease in pro-inflammatory cytokines and collection of myofibroblast | (71) |
| Menstrual blood-derived  endometrial stem cells | EVs | Fibrosis | Lung tissue | ↓Hydroxyproline, ↓MDA, ↑Let-7 | Decrease DNA damage, ROS and collagen deposition | (72) |
| BMDMC | BMDMC | Sepsis | - | Decreased caspase-3, IL-6, IL-1β, VEGF, and TGF-β | Inhibited inflammations, attenuated lung injury, enhanced IL-10 levels in lung tissues of septic mice, thus improving repair of endothelium and epithelium | (73) |
| BMDMC | BMDMC | ALI | - | Reduced the levels of IL-6, IL-10, IGF, PDGF, and TGF-β | Improved lung injury, modulated inflammatory and fibrogenic responses | (74) |
| BMDMC | BMDMC | ALI | - | Decreased inflammatory cytokines | Improved lung injury and alleviate the function of pulmonary cells | (75) |
| HA-MSC | EVs | Fibrosis | Lung tissue | ↓TGF-β, ↓TNF-α, ↓IL-1β | Decrease in alveolar wall thickness; enhance anti-inflammatory cytokines and collagen deposition | (76) |
| Amnion epithelial cells | EVs | Fibrosis | Lung tissue | ↑CTNNB1, ↑BMP4, ↑BMPR1 | Decrease CD4 + T and collagen deposition | (77) |
| BM-MSCs | EVs | ALI | LMVECs | ↑VE-cadherin, ↑ZO-1 | Diminish of microvascular permeability | (78) |
| BM-MSCs | Exosome | ALI | Alveolar/ macrophages | IL-10; TGF-beta | Increased immunosuppressive phenotype | (79) |
| EPC | EVs | Hypoxia-reoxygenation injury model | ECs | miR-126 | Improved function of ECs | (80) |
| BM-MSC | EVs | ARDS | Lung tissue | ↑Runx1p66/p52, ↑TβRI/Alk5 | Decrease in perivascular area | (81) |
| BM-MSC | EVs | ALI | Lung tissue | ↓TNF-α, ↓CXCL10, ↑ IL-10 | Decrease in lung endothelial cell apoptosis | (82) |
| BM-MSC | EVs | ALI | Lung tissue | ↓Apoptosis | Decrease in alveolar wall thickness; enhance anti-inflammatory cytokines and collagen deposition | (83) |
| EPC | EPC | ALI | ECs and  other lungs  cells | Reduced levels of IL-1β, ROS, NO, and enhanced amount of IL-10 and VEGF | Improved function of ECs in acute lung injury | (84) |
| EPC | EPC | ALI | ECs | Decreased endothelin1 and iNOS; Enhance IL-10 | Improved function of ECs and increased survival acute lung injury | (85) |
| S. aureus | EVs | Pneumonia | Lung tissue | ↓IL-1β, ↓IL-6, ↑IL-17, ↑IL-4 | Decrease in pro-inflammatory  cytokines; enhance anti-inflammatory cytokines | (86) |
| BM-MSC | EVs | Pneumonia: | Lung tissue/ Alveolar epithelial cells | ↓MIP-2, ↑KGF, ↓TNF-α | Decrease in pro-inflammatory  cytokines; enhance anti-inflammatory cytokines | (87) |
| BM-MSC | EVs | ARDS | Lung tissue | ↓MIP-2 | Decrease in pro-inflammatory  cytokines; enhance anti-inflammatory cytokines | (88) |
| MAPC | MAPC | ALI | - | Decreased the levels of TNF-α, IL-1β, and IFN-γ | Inhibition of innate immune responses | (89) |
| iPSC | iPSC | ALI | PMN | Decreased the level of  VCAM-1 and MIP-2, TNF-α, IL-6 | Improved lung injury, attenuated the effects of neutrophils in induction of inflammatory response in lung, attenuated LPS-induced injury | (90) |
| iPSC | iPSC or  iPSC CM | ALI | PMN | Enhances GRK2 activity, and decreased the level of  CXCR2 | Improved lung injury, reduced neutrophil chemotaxis in endotoxin-associated ALI | (91) |
| Neutrophils loaded with  piceatannol | EVs | ALI | Lung tissue | ↓TNF-α, ↓IL-6, ↓MPO | Decrease in leukocytes and neutrophils in alveolar fluid | (92) |
| BM-MSC | EVs | ALI | Lung tissue | ↓MIP-2 | Decrease in alveolar wall thickness; enhance anti-inflammatory cytokines and collagen deposition | (93) |
| Urine-derived pluripotent stem  cells | EVs | ALI | Lung tissue | ↓ICAM-1, ↓MPO | Decrease in pro-inflammatory cytokines | (94) |
| iPSC-MSC | Released SCF | ALI | Bronchial  epithelial  cells | Decreased CINC-1 | Improved the function of lung airway cells | (95) |
| iPSC | iPSC CM | VILI | - | Suppressed PI3K/Akt pathway | Improved lung injury | (96) |
| ESC-AECII | ESC derived AECII | ALI | AEC I | Collagen deposition | Attenuated lung injury | (97) |
| BMDMC | BMDMC | Emphysema | - | Decreased the levels  of KC, MIP-2, and IFN-γ | Inhibited inflammations and apoptosis; improved pulmonary function | (98) |
| HA-MSC | EVs | ALI | Lung tissue | ↓TNF-α, ↓IL-1β, ↓IL-6, ↑IL-10 | Decrease pro-inflammatory  cytokines and enhance anti-inflammatory cytokines | (99) |
| Lung spheroid cells | EVs | Fibrosis | Lung tissue | ↑AQP5, ↑vWF, ↓αSMA, | Enhance alveolar repair and decrease collagen  deposition | (100) |
| BM-MSC | EVs | ARDS | Lung tissue | ↓TNF-α, ↓IL-6, ↓KC, ↓VEGF,  ↓TGF- β | Decrease neutrophil and  macrophage recruitment in alveolar fluid | (101) |
| PMVECs | EVs | ALI | Lung tissue | ↓IL-6, ↓IL-1β, ↓TNF-α, ↓F-actin, ↓MLC | Decrease in alveolar wall thickness and pro-inflammatory cytokines and enhance anti-inflammatory cytokines | (102) |
| UC-MSC | EVs | ALI | Lung tissue | ↓TNF-α, ↓IL-6, ↑HGF, ↑c-Met | Decrease in pro-inflammatory  cytokines; enhance anti-inflammatory cytokines; diminishing of alveolar epithelial apoptosis and collagen deposition | (103) |
| BM-MSC | EVs | ALI | Lung tissue | ↓MMP-9 ↑SP-C ↓TNF-α, ↓IL-1β | Restoration of respiratory function; enhancement of alveolar epithelial  surfactant | (104) |
| UC-MSC | EVs | ALI | Lung tissue | ↑eNOS, ↓TNF-α | enhance anti-inflammatory cytokines; enhancement of  alveolar epithelial surfactant | (105) |
| Umbilical cord EPC (rich in  miR-126) | EVs | ALI | Lung tissue | ↓TNF-α, ↓IL-6, ↓IL-1β, ↓IFN-γ | Decrease hyaline membrane formation and pro -inflammatory cytokines | (106) |
| UC-MSC | EVs | ALI | Lung tissue | ↓TNF-α, ↓IL-1β, ↓IL-6, ↑IL- 10 | Enhance anti-inflammatory cytokines | (107) |
| Whole blood | EVs | Fibrosis | Lung tissue | ↓Hydroxyproline | Decrease in alveolar wall thickness; enhance anti-inflammatory cytokines and collagen deposition | (108) |
| Pulmonary microvascular  endothelial cells | EVs | ALI | Lung tissue | ↓TNF-α, ↓IL-1β, ↓IL-6 | Decrease in alveolar wall thickness; enhance anti-inflammatory cytokines and collagen deposition | (109) |
| BM-MSC | EVs | ARDS | Lung tissue | ↓TNF-α | Decrease in alveolar wall thickness; enhance anti-inflammatory cytokines and collagen deposition | (79) |
| BM-MSC | EVs | Fibrosis | Lymphatic endothelial cells | ↓αSMA, ↓Col IA1, ↓Col III A1 | Decrease in pro-inflammatory  cytokines; enhance anti-inflammatory cytokines | (67) |
| BM-MSC | EVs | ALI | Lung tissue | ↓MIP-2 | Decrease in pro-inflammatory  cytokines; enhance anti-inflammatory cytokines | (110) |

EPC: Endothelial progenitor cell, ECs: epithelial cells, HSAEpC: Human Small Airway Epithelial Cells, LAP: lung alveolar epithelial, EnCs; Endothelial cells, CLP: Cecum Ligation and Puncture, VEGF: Vascular endothelial growth factor, MAPC: multipotent adult progenitor cell, BMDMC: Bone marrow-derived mononuclear cell, iPSC-CM: Induced pluripotent stem-cell derived cardiomyocytes; PMN: polymorphonuclear leukocytes, VILI: ventilator-induced lung injury, SCF: Stem cell factor, CINC-1: cytokine-induced neutrophil chemoattractant-1, ESC-AECII: ESC derived alveolar type II epithelial cell, ALI: acute lung injury, DCs: dendritic cells. HA-MSC: human adipose-derived mesenchymal stem cells, PMVECs: Pulmonary microvascular endothelial cells; ARDS: Acute respiratory distress syndrome,

LMVEC: Lung microvascular endothelial cell,

Supplementary Table 2. Summary of studies which reported the role of extracellular vesicles in the treatment of osteoarthritis.

| Cell origin | Type of secreted vesicle | Disease | Target cells or tissues | Molecular mechanism | Biological effect & therapeutic applications | Reference |
| --- | --- | --- | --- | --- | --- | --- |
| BM-MSC | Exosomes | In vitro | Chondrocytes | miR-138-5p | Increase the proliferation and  decrease apoptosis | (113) |
| MSCs | EVs | In vitro | Chondrocytes | miR-let7c | The anti-fibrotic function | (114) |
| hSM-MSCs | Exosomes | In vitro | Chondrocytes | miR-140-5p | Increase regeneration of cartilage | (115) |
| BM-MSCs | Exosome | Osteoarthritis | Chondrocytes | miR-320c and  miR-92a–3p | Enhance regeneration of cartilage | (111) |
| hSM-MSCs | Exosomes | Osteoarthritis | Chondrocyte | - | Increase the proliferation and function of chondrocyte | (112) |
| MSCs | EVs | In vitro | Chondrocytes | hsa-miR-10a-5p, hsa-miR-29a-3p,  hsa-miR-127-3p, hsa-miR-486-5p | Adjust the biological activity of EVs | (116) |
| MSCs | Exosomes | In vitro | Chondrocytes | miR-21 | Decrease of apoptosis | (117) |
| BM-MSC | Exosomes | In vitro | Chondrocytes | miR-124 | Increase cortical neural | (42) |
| BM-MSCs | Exosome | Osteoarthritis | Chondrocytes | ADK and NDKD pathway | Reconstruction in cell metabolism and cartilage | (118) |
| BM-MSCs/ AT-MSCs | Exosome/ conditioned medium | In vitro | Chondrocytes | - | Decrease secretion of inflammatory cytokines | (27, 119) |
| MSCs | Exosomes | In vitro | Chondrocytes | miR 146b | Decrease tumor mass | (120) |
| AD-MSCs | Exosomes | In vitro | Chondrocytes | miR-146a | Decrease myocardial damage | (121) |

human synovial membrane-derived mesenchymal stem cells (hSM-MSCs); Bone marrow mesenchymal stem cells (BM-MSCs); adipose tissue mesenchymal stem cells (AT-MSCs), nucleoside-diphosphate kinase-dependent pathways (NDKD pathways); adenylate kinase (ADK).

Supplementary Table 3. Summary of studies which reported the role of extracellular vesicles in the treatment of colitis.

| Cell origin | Type of secreted vesicle | Disease | Target cells or tissues | Molecular mechanism | Biological effect & therapeutic applications | Reference |
| --- | --- | --- | --- | --- | --- | --- |
| BM-MSCs | EVs | Colitis | - | miR-200b | Reduce fibrosis in colitis | (122) |
| UC-MSCs | Exosome | Colitis | macrophages | IL-10/IL-7 | Decrease secretion of inflammatory cytokines | (123) |
| BM-MSCs | EVs | Colitis | leukocytes | NF-kB-p65 | Decrease secretion of inflammatory cytokines | (124) |
| BM-MSCs | Exosome | Colitis | epithelial cell | IL-10; IDO-1/KYN | Decrease expression of  Ubiquitin, K48, K63, and FK2 | (125) |
| BM-MSCs | EVs | Colitis | epithelial cell | MPO, MDA, SOD,  GSH/caspase-3,-8  and -9 | Reduce oxidative stress and apoptosis | (124) |

Supplementary Table 4. Summary of studies which reported the role of extracellular vesicles in the treatment of cardiac disorders.

| Cell origin | Type of secreted vesicle | Disease | Target cells or tissues | Molecular mechanism | Biological effect & therapeutic applications | Reference |
| --- | --- | --- | --- | --- | --- | --- |
| BM-MSCs | Exosome | Myocardial  ischemia/reperfusion injury | Cardiomyocytes | PI3K/Akt | Modulate contractility and decrease infarct size | (126, 129) |
| Mouse iPS | EVs | Myocardial infarction | - | - | Decrease pro-inflammatory monocytes | (130) |
| Human ESC | EVs | Myocardial infarction | Cardiomyocyte | Targeting  miR-497 by | Decrease apoptosis in myocytes | (131) |
| Human ESC | EVs | Myocardial infarction | Cardiomyocyte | Gene regulation and  DNA repair | Decrease scar size  and cardiomyocyte apoptosis | (132) |
| Mouse ESC | EVs | Myocardial infarction | Cardiomyocyte | Regulation of CPC | Increase cardiomyocyte proliferation | (133) |
| BM-MSCs | Exosome | Myocardial  ischemia/reperfusion injury | Cardiomyocytes | AMPK/mTOR and  Akt/mTOR | Inhibition of apoptosis /enhancement in autophagy/ decrease infarct size | (134) |
| BM-MSCs | Exosome | Myocardial  ischemia/reperfusion injury | Cardiomyocytes | N-SMase/miR-210 | Increase survival/decrease scar size/improve cardiac function | (135) |
| BM-MSCs | Exosome | Myocardial  ischemia/reperfusion injury | Endothelial cells | SDF-1/miR-132 | Increase angiogenesis in the infarct zone | (127, 136) |
| CPCs | EVs | Myocardial infarction | Cardiomyocyte | Activation of  endoglin in  ECs | Decrease infarct size | (137) |
| CPCs | EVs | Myocardial infarction | Cardiomyocyte | Decreased levels  of collagen I and III,  vimentin | Decrease infarct size | (138) |
| Human iPS | EVs | Myocardial infarction | - | miR-146a-3p,  miR-132, and  PAPP-A | Enhance cardiac function | (139) |
| Human iPS | EVs | Myocardial infarction | - | Regulation of  gene expression | Enhance cardiac function | (140) |
| BM-MSCs | Exosome | Ischemia/reperfusion  injury | Cardiomyocytes/macrophages | IL-10/VEGF | Inhibit apoptosis in cardiomyocyte/ decrease secretion of inflammatory  Cytokines in macrophage/ improve infarct zone | (141) |
| BM-MSCs | Exosome | Myocardial  ischemia/reperfusion injury | Cardiomyocytes | Bcl-2, Bax, caspase-3;  Wnt/-catenin | Inhibition of apoptosis/ decrease infarct size | (142) |
| CDCs | EVs | Myocardial infarction | - | - | Decrease infarct size  and enhance cardiac function | (143) |
| CDCs | EVs | Myocardial infarction | - | Regulation of  gene expression | Decrease microvascular occlusion, infarct size | (144) |
| CDCs | EVs | Myocardial infarction | - | - | Increase cardiac function | (144) |
| CPCs | EVs | Myocardial infarction | - | Regulation of  gene expression | Increased cardiac function, wall thickness | (145) |
| BM-MSCs | Exosome | Myocardial  ischemia/reperfusion injury | Cardiomyocytes | miR-210 and  miR-125b-5p | Decrease infarct size | (135, 146) |
| BM-MSCs | EVs | Cardiomyocyte contractility | - | miR-21p | Enhance contractility | (147) |
| BM-MSCs | EVs | Cardiac infarction | Cardiac cells | miR-22 | Reduce cardiac fibrosis | (148) |
| UC- MSCs | EVs | Myocardial infarction | - | - | Safety | (149) |
| UC- MSCs | EVs | Myocardial infarction | - | - | Decrease cardiomyocyte apoptosis | (59) |
| UC- MSCs | EVs | Myocardial infarction | Cardiomyocyte | Upregulation of  Smad7 | Enhance cardiomyocyte survival | (150) |
| Cardiac MSCs | EVs | Myocardial infarction | Cardiomyocyte | - | Increase cardiac function, Angiogenesis | (151) |
| CDCs | EVs | Myocardial infarction | Cardiomyocyte | - | Decreased infarct size and  leukocyte infiltration | (152) |
| BM-MSCs | EVs | Myocardial infarction | - | miR-210 | Protection against cardiac cells | (135) |
| BM-MSCs | EVs | Sepsis | Cardiac cells | miR-223 | Repair and recovery of cardiac cells | (153) |
| BM-MSCs | EVs | Ischemic cardiomyopathy | - | miR-21 | Decrease oxidative injury | (117) |
| BM-MSCs | EVs | Myocardial infarction | - | Decrease  expression of  pro-apoptotic  genes | Reduce infarct size | (154) |
| BM-MSCs | EVs | Myocardial infarction | - | miR-125b | Increase cardiac function | (146) |
| BM-MSCs | EVs | Myocardial infarction | Cardiomyocyte | miR-24 | Decrease infarct size  And enhance cardiomyocyte apoptosis | (155) |
| AD-MSCs | EVs | Myocardial infarction | Macrophage | S1P/SK1/S1PR1  activation | Decrease fibrosis, apoptosis;  Increase anti-inflammatory  effects | (156) |
| END-MSCs | EVs | Cardiac infarction | - | miR-21 | Anti-apoptotic and anti-angiogenic effects | (157) |
| PD-MSCs | EVs | Ischemia | - | Jagged1 | Enhances angiogenesis | (158-160) |
| BM-MSCs | EVs | Myocardial infarction | Macrophages | Inhibition of  TLR4 by miR-182 | Decrease infarct size;  moderate inflammation | (108) |
| BM-MSCs | EVs | Myocardial infarction | Macrophages | Suppression of  NF-κB and  regulation of  AKT1/AKT2 | Decrease inflammation and cardiomyocyte apoptosis;  Anti-inflammatory effects | (161) |
| BM-MSCs | EVs | Myocardial infarction | - | - | Increase cardiac function, inflammation, and vascularization;  Decrease infarct size | (162) |
| BM-MSCs | EVs | Myocardial infarction | Endothelial cells | lncRNA H19 and  miR-675 | Increase angiogenesis and cardiac function; Decreased cardiomyocyte apoptosis | (163) |
| BM-MSCs | EVs | Myocardial infarction | Endothelial cells | miR-210 and  Efna3 gene  suppression | Increase cardiac function, angiogenesis, and fibrosis | (164) |
| UC- MSCs | EVs | Myocardial infarction | Endothelial cells | PDGF-D | Change the biology of EnCs and induce migration and tube formation | (128, 165) |
| UC- MSCs | EVs | Hypoxia-ischemia | - | miR-let-7e, miR-let-7a | Anti-apoptotic effect | (166, 167) |
| hiPSC-CM | EVs | Myocardial infarction | Endothelial cells | Pro-angiogenic growth factors | Enhances angiogenesis | (168) |
| BM-MSCs | EVs | Myocardial infarction | - | - | Increase cardiac function;  Decrease fibrosis  and inflammation | (169) |

Bone marrow mesenchymal stem cells (BM-MSCs); umbilical cord mesenchymal stem cells (UC-MSCs); endometrial mesenchymal stem cells (END-MSCs); dental pulp mesenchymal stem cells (PD-MSCs), hiPSC-CM: human induced pluripotent stem cell-derived cardiomyocytes, CDCs: cardiosphere-derived cells, CPCs: cardiac progenitor cells, ESC: embryonic stem cells

Supplementary Table 5. Summary of studies which reported the role of extracellular vesicles in the treatment of spinal cord injury.

| Cell origin | Type of secreted vesicle | Target cells or tissues | Molecular mechanism | Biological effect & therapeutic applications | Reference |
| --- | --- | --- | --- | --- | --- |
| BM-MSCs | EVs | Pericytes | NF-kB-p65 | Decrease migratory potential  of pericytes/enhance  the integrity of BSCB | (170) |
| BM-MSCs | Exosome | Neurons | miR-133b/Erk1/2  and Stat-3 | Increase the regeneration of axons | (171) |
| BM-MSCs | Exosome | N1 astrocytes | L-10/NF-kB-p65 | Decrease secretion of  inflammatory cytokines | (172, 173) |
| BM-MSCs | Exosome | Macrophages | IL-10/NF-kB-p65 | Convert M1 macrophage phenotype to M2 macrophage phenotype | (159, 174) |

Supplementary Table 6. Summary of studies which reported the role of extracellular vesicles in the treatment of other disorders.

| Cell origin | Type of secreted vesicle | Disease | Target cells or tissues | Molecular mechanism | Biological effect & therapeutic applications | Reference |
| --- | --- | --- | --- | --- | --- | --- |
| BM-MSCs | EVs | Middle cerebral artery occlusion | - | miR-133b | Increase neurite outgrowth | (175) |
| EMB- MSCs | EVs | Angiogenesis | EnCs | miR-30b | Increase angiogenesis | (179) |
| AD-MSCs | EVs | Erectile dysfunction in diabetes | - | miR-126, miR-130a, miR-132, miR-let7b, miR-let7c | Improve erectile function | (180) |
| BM-MSCs | EVs | Renal fibrosis | - | miR-294, miR-133b-3p | Induce EMT | (181) |
| BM-MSCs | EVs | Stroke | - | miR-133b | Neuroprotective effects | (182) |
| BM-MSCs | EVs | Alzheimer’s disease | - | miR-21 | Prevent degeneration of neurons | (176) |
| BM-MSCs | Exosomes | Alzheimer’s disease | N2a cells | A beta peptide | Reduce A beta peptides | (183) |
| BM-MSCs | Exosomes | Neurodegenerative  disease | Neurons | - | Improve neurons function | (184) |
| BM-MSCs | EVs | Hindlimb ischemia | EnCs | miR-210-3p, VEGF | Increase angiogenesis | (185) |
| BM-MSCs | EVs | Acute kidney injury | - | CCR2 | Repair and recovery of an injured cell | (186) |
| BM-MSCs | EVs | Kidney transplantation | - | miR-146a | Enhance graft survival | (178) |
| BM-MSCs | EVs | Systemic sclerosis | Osteocyte | miR-151-5p | Enhance osteogenesis | (187) |
| BM-MSCs | Exosome | Glaucoma | RGCs | - | Enhance proliferation and survival of RGCs/ Improve clinical condition in glaucoma | (177) |
| BM-MSCs | EVs | Intervertebral disc degeneration | NPCs | miR-21 | Suppress apoptosis of NPCs | (188) |
| PL-MSCs | EVs | Hindlimb ischemia | - | VEGF/miR-126 | Enhance angiogenesis | (189) |
| UC- MSCs | EVs | CIRTECs | Renal tubular  epithelial cells | - | Protect against CIRTECs | (190) |
| UC- MSCs | EVs | Sepsis | Macrophages | miR-146a | Diminish inflammatory cytokines | (19) |
| BM-MSCs | Exosome | EAU | DCs, Th1, Th17 cells | IL-10; IDO-1/KYN | Decrease function of DCs; and production of Th1 and Th17-related cytokines and Improve clinical condition in EAU | (191) |
| BM-MSCs | Exosome | EAU | Neutrophils, NK cells,  macrophages and T cells | MCP-1; CCl21 | Decrease number of inflammatory  cells/Improve clinical condition in EAU | (192) |
| BM-MSCs | EVs | Skeletal muscle regeneration | Muscles cell | miR-494 | Enhance angiogenesis | (193) |
| BM-MSCs | EVs | Acute kidney injury | Renal cells | CCNB1, CDK8, CDC6 | Repair and recovery of an injured cell | (194) |
| EMB- MSCs | EVs | Osteochondral defect | Cartilage cells | CD73 | Repair and recovery of cartilage cells | (195) |
| UCD-MSCs | Exosome | Sly syndrome | Keratocytes | Beta-glucuronidase-induced degradation of GAGs | Increase the degradation of GAGs and improve the clinical condition of Sly syndrome | (196) |
| BM-MSCs | Exosome | Laser-induced  retinal injury | Macrophages | MCP-1 | Decrease activation of inflammatory macrophages and the number of photoreceptor cells | (197) |
| UC- MSCs | EVs | Skin defect | Myofibroblast | miR-21, miR-23a, miR-125b, miR-145 | Reduce scar formation by a change in the development of myofibroblasts | (198) |
| UC- MSCs | EVs | Skin defect in diabetes | - | miR-let-7b | Increase healing of cutaneous injuries | (199) |
| UCD-MSCs | Exosome | NIIP | Neurons; glial  cells | BDNF,  GDNF/IL-10 | Decrease stimulation of neurons, improve the clinical condition of NIIP pain | (200) |
| AT-MSCs | EVs | Angiogenesis | EnCs | miR-125a | Promote angiogenic capacity of EnCs | (201-203) |
| BM-MSCs | EVs | Leukocyte activation | Leukocytes | COX2/PGE2 | Inhibit secretion of inflammatory cytokines | (204) |
| BM-MSCs | EVs | Optic nerve crush | RGCs | Argonaute-2 | Enhance regeneration of RGCs axons | (205) |
| BM-MSCs | Exosomes | Wound healing | - | Decreasing TNF-α and enhancing IL-10 | Wound healing | (206) |
| UC-MSCs | Exosomes | Wound healing | - | Decreasing TNF-α, IL-1, TLR4 and increasing IL-10 | Wound healing and improvement of injuries | (207) |

Bone marrow mesenchymal stem cells (BM-MSCs); adipose tissue mesenchymal stem cells (AT-MSCs); umbilical cord mesenchymal stem cells (UC-MSCs); embryonic mesenchymal stem cells (EMB-MSCs); interleukin (IL)-10; Kynurenine (KYN); C-C chemokine receptor type 5 (CCR5); transforming growth factor beta (TGF-β); T regulatory cells (Tregs); indoleamine 2,3 dioxygenase-1 (IDO-1); fibroblast growth factor 2 (FGF-2); keratinocyte growth factor (KGF); dendritic cells (DCs); chronic obstructive pulmonary disease (COPD); Kynurenine (KYN), malondialdehyde (MDA); indoleamine 2,3 dioxygenase-1 (IDO-1); protein kinase B (Akt); superoxide dismutase (SOD); nuclear factor-kB (NF-kB); glutathione (GSH); phosphoinositide-3-kinase (PI3K); monocyte chemoattractant protein 1 (MCP-1); vascular endothelial growth factor (VEGF); mammalian target of rapamycin (mTOR); experimental autoimmune uveitis (EAU); glycosaminoglycans (GAGs); AMP-activated protein kinase (AMPK); retinal ganglion cells (RGCs); brain-derived neurotrophic factor (BDNF); platelet-derived growth factor (PDGF); natural killer (NK) cells; NIIP; nerve-injury induced pain, blood-spinal cord barrier (BSCB); IL-1 receptor antagonist (IL-1Ra); glial cell line-derived neurotrophic factor (GDNF); PL-MSCs; placenta mesenchymal stem cells, CIRTECs; Cisplatin-injured renal tubular epithelial cells, EMT; epithelial mesenchymal transition, RGCs; Retinal ganglion cells, NPCs; nucleus pulposus cell.
